# Long‐distance pollen and seed dispersal and inbreeding depression in *Hymenaea stigonocarpa* (Fabaceae: Caesalpinioideae) in the Brazilian savannah

**DOI:** 10.1002/ece3.4253

**Published:** 2018-07-13

**Authors:** Marcela A. Moraes, Thaisa Y. K. Kubota, Bruno C. Rossini, Celso L. Marino, Miguel L. M. Freitas, Mario L. T. Moraes, Alexandre M. da Silva, Jose Cambuim, Alexandre M. Sebbenn

**Affiliations:** ^1^ Faculdade de Engenharia de Ilha Solteira/UNESP Ilha Solteira SP Brazil; ^2^ Instituto de Biociências de Botucatu/UNESP Botucatu SP Brazil; ^3^ Instituto Florestal de São Paulo São Paulo SP Brazil

**Keywords:** ex situ conservation, microsatellite loci, mixed mating system, neotropical tree

## Abstract

*Hymenaea stigonocarpa* is a neotropical tree that is economically important due to its high‐quality wood; however, because it has been exploited extensively, it is currently considered threatened. Microsatellite loci were used to investigate the pollen and seed dispersal, mating patterns, spatial genetic structure (SGS), genetic diversity, and inbreeding depression in *H. stigonocarpa* adults, juveniles, and open‐pollinated seeds, which were sampled from isolated trees in a pasture and trees within a forest fragment in the Brazilian savannah. We found that the species presented a mixed mating system, with population and individual variations in the outcrossing rate (0.53–1.0). The studied populations were not genetically isolated due to pollen and seed flow between the studied populations and between the populations and individuals located outside of the study area. Pollen and seed dispersal occurred over long distances (>8 km); however, the dispersal patterns were isolated by distance, with a high frequency of mating occurring between near‐neighbor trees and seeds dispersed near the parent trees. The correlated mating for individual seed trees was higher within than among fruits, indicating that fruits present a high proportion of full‐sibs. Genetic diversity and SGS were similar among the populations, but offspring showed evidence of inbreeding, mainly originating from mating among related trees, which suggests inbreeding depression between the seed and adult stages. Selfing resulted in a higher inbreeding depression than mating among relatives, as assessed through survival and height. As the populations are not genetically isolated, both are important targets for in situ conservation to maintain their genetic diversity; for ex situ conservation, seeds can be collected from at least 78 trees in both populations separated by at least 250 m.

## 1 INTRODUCTION

Tropical forests around the world have experienced extensive fragmentation, resulting in tree populations that are spatially isolated in small forest fragments and individuals scattered throughout landscapes interspersed with pastures, agricultural areas, highways, and cities. This is especially true for the Brazilian savannah, where the biome has been significantly deforested (Sano, Rosa, Brito & Ferreira, 2007). As clear‐cutting has resulted in the loss of many tree populations and their genetic material, urgent strategies for the in situ and ex situ conservation of remnant tree populations throughout the savannah biome are needed. Information about pollen dispersal patterns is required to identify and understand if populations and individuals that are spatially isolated in this landscape are also genetically isolated.

Habitat fragmentation can result in negative impacts on the remaining tree populations; it disrupts reproductive processes and decreases pollen and seed flow due to spatial isolation, resulting in decreased genetic diversity and effective population size and increased intrapopulation spatial genetic structure (SGS) and inbreeding, affecting subsequent generations (Degen & Sebbenn, 2014; Finger et al., 2012; Ismail et al., 2012). The spatial isolation of trees may decrease the reproductive success because plants may receive fewer visitors to flowers due to a decline in the richness and abundance of pollinator vectors, modifications to species composition, and limitations to movement among populations (Goverde, Schweizer, Baur & Erhardt, 2002). Fragmentation can, thus, modify the activity of pollinators by reducing the density of potential food resources and increasing the distance between those resources (Sih & Baltus, 1987). As plant densities decline, animal pollinators are less likely to shift from one plant to another because of the increased costs of foraging. Furthermore, pollinators foraging on self‐compatible plants for longer periods of time can increase the probability of selfing (Karron, Holmquist, Flanagan & Mitchell, 2009). A review of mating systems across 27 plant species in undisturbed versus disturbed populations confirmed the expectation of increased self‐fertilization in disturbed plant populations (Eckert et al., 2010).

For a variety of reasons, detecting the effects of forest fragmentation is difficult. Tropical trees are generally long‐living species, with many adults in remnants from prefragmentation stages that present overlapping generations, undergo regular long‐distance gene flow, and may present adaptive mechanisms for species survival, such as a mixed mating system, thus producing seeds by both outcrossing and self‐fertilization (Lower, Cavers, Boshier, Breed & Hollingsworth, 2015). To study the effects of fragmentation, it is important to use samples from different ontogenic stages, including adults, juveniles, and seeds, as the genetic effects of forest fragmentation may be detected only in the new generations.

Advances in molecular genetic markers, statistical analyses, and software development have enabled the detailed investigation and understanding of processes such as mating systems, gene dispersal patterns, and spatial genetic structure and the quantification of genetic diversity, genetic structure, and inbreeding. Parentage analysis (paternity and maternity) has been used extensively to study gene dispersal in tree populations in a wide range of environments, including continuous, fragmented, and logged forests, isolated trees in pastures, and seed orchards (Burczyk, DiFazio & Adams, 2004; Ellstrand, 2014; Oddou‐Muratorio & Klein, 2008; Smouse & Sork, 2004). In such analyses, it is important to differentiate between the realized and effective gene flow because deterministic factors such as natural selection and stochastic factors such as random mortality, predation, and disease can come into play between the seed and juvenile (regenerant) stages, and many seeds never reach the juvenile and/or adult stages. Paternity and maternity analyses based on samples of established regenerants allow us to assess the realized pollen and seed dispersal, whereas paternity analysis based on open‐pollinated seeds represents the effective pollen dispersal or the results of fertilization (Burczyk et al., 2004). Greater realized pollen flow than seed flow in fragmented populations has been reported for animal‐pollinated and animal‐ and wind‐dispersed seeds of tropical trees (Baldauf et al., 2014; Gaino et al., 2010; Ismail et al., 2017; Sebbenn et al., 2011).

Due to the high rate of deforestation in the Brazilian savannah biome, studies on the deciduous and monoecious tree *Hymenaea stigonocarpa* Mart. ex Hayne (Fabaceae: Caesalpinioideae) in the region are needed. In the São Paulo and Mato Grosso do Sul states, *H. stigonocarpa* is currently only found in forest fragments and as isolated trees in pastures; therefore, plans for its in situ and ex situ conservation are urgent. In Brazil, the species occurs between 3°30′S and 22°40′S, and as such, it is restricted to the savannah habitat in the central and south‐east regions of the country (Carvalho, 2006). *Hymenaea stigonocarpa* trees can reach up to 29 m in height and 50 cm in diameter at breast height (dbh). At least four different bat species pollinate *H. stigonocarpa* flowers, including *Glossophaga soricina*,* Platyrrhinus lineatus*, and *Carollia perspicillata* (Gibbs, Oliveira & Bianchi, 1999), and both agoutis and birds disperse the fruits and seeds (Ramos, Lemos‐Filho & Lovato, 2009). The economic importance of the species is related to its multiple uses; its wood can is used for construction; a yellow dye extracted from its bark is used in various sectors; and its fruits are edible. *Hymenaea stigonocarpa* is also important for fauna, serving as food for parakeets, parrots, howler monkeys, rodents, small wolves, and insects (Botelho, Ferreira, Malavasi & Davide, 2000), which are also likely seed dispersers. The species is self‐compatible, with postzygotic selection in self‐pollinated flowers (Gibbs et al., 1999) and a mixed mating system (Moraes & Sebbenn, 2011). The species has been indicated for use in the recovery of degraded savannah areas (Moraes & Sebbenn, 2011). While the mating system of *H. stigonocarpa* is relatively well understood, there is limited information about gene flow among trees occurring in pastures and forest fragments (Moraes & Sebbenn, 2011). Estimates of outcrossing, correlated mating, inbreeding, and pollen and seed dispersal distance are vital to inform conservation strategies for populations in highly fragmented environments and to calculate the number of seed trees required for seed collection in ex situ conservation and environmental reforestation (Sebbenn, 2006).

Due to the fact that in the São Paulo and Mato Grosso do Sul states, *H. stigonocarpa* is currently only found in forest fragments and as isolated trees in pastures, we investigated the effects of forest fragmentation on the genetic structure of two of the remaining populations in this region, where the species populations that remain are not continued in natural forests and are only found in fragmented populations. We used microsatellite loci to assess the genetic diversity, inbreeding, intrapopulation SGS, mating system (hierarchically within and among fruits), and seed and pollen dispersal of *H. stigonocarpa* populations occurring in a forest fragment population and in a pasture. We also sought to determine the number of seed trees needed for seed collection in these sites for ex situ conservation and environmental reforestation. We addressed the following questions: (a) Is there gene flow between the pasture and the forest fragment? (b) What are the distance and patterns of pollen and seed dispersal in the populations? (c) Are there differences in the rates of selfing and mating among relatives, and is there a paternity correlation between trees in the pasture and the forest fragment? (d) Is the paternity correlation lower among fruits than within fruits? (e) Are the levels of effective size variance lower and inbreeding higher in open‐pollinated seeds collected from the pasture than in seeds collected from trees in the forest fragment? (f) Do selfing and mating among related trees produce inbreeding depression?

## 2 MATERIALS AND METHODS

### 2.1 Study site and sampling

**Figure 1:**
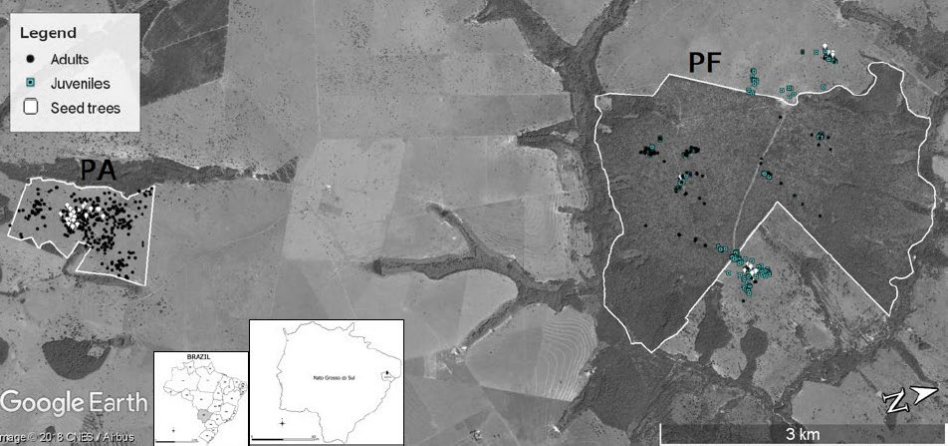
Spatial distribution of *Hymenaea stigonocarpa* trees in the pasture (PA) and forest fragment (PF)

The studied savannah landscape is characterized by high levels of anthropogenic disturbances. It consists of large (approximately 2,523 ha), extended pastures and sugarcane and eucalyptus plantations, interspersed with small forest fragments and isolated *H. stigonocarpa* trees in the pastures. The area (20°07′S, 51°44′W, altitude of 373 m) is located near highway MS444, in the municipality of Inocência, Mato Grosso do Sul State, Brazil. The climate is tropical with a dry winter, a humid summer, an average annual rainfall of 1,232 mm, and an average temperature of 24.5°C. In this region, much of the savannah was cut down between 1970 and 1980 for livestock production. The study was carried out in two populations (PA and PF) located approximately 5 km apart (Figure 1), in which all adult trees and juveniles were mapped (GPS Garmin model GPSmap 62 sc), measured for dbh for adults or total plant height (*H*) for juveniles, sampled, and genotyped. The PA population (109.12 ha) consists of aggregated or isolated trees in a pasture (*Brachiaria* sp.), with an estimated density of 3.29 trees/ha. From this population, we sampled 359 adult trees (dbh = 43.9 ± 84.2 cm, mean ± standard deviation; distance ranging from 1 to 528 m, mean of 466 m). The PF population is located within a large forest fragment (666.7 ha) with a population density of 0.17 trees/ha. Within PF, trees occur in small, spatially isolated groups. From the PF fragment, we sampled 111 adult trees (dbh = 24.5 ± 15.3 cm; distance ranging from 3 to 2,727 m, mean of 1,136 m) and 219 juveniles (*H* = 1.34 ± 1.36 cm; distance ranging from 1 to 2,641 m, mean of 1,282 m), representing nonreproductive plants. We also collected and genotyped open‐pollinated seeds from 20 seed trees in the PA population and 15 seed trees in the PF population; 30 seeds per tree from different fruits were included the sample, resulting in a total of 600 PA seeds and 450 PF seeds. Seeds were identified by mother (family) and fruit origin for the hierarchical paternity analysis within and among fruits. To support the ex situ conservation of the populations and to investigate the inbreeding depression, we germinated seeds and used the resulting offspring to establish a provenance and progeny test in the Fazenda de Ensino, Pesquisa e Extensão, UNESP Ilha Solteira Campus (FEIS/UNESP), located in Selvíria, Mato Grosso do Sul State, using an unbalanced random block design comprising two provenances (PF and PA populations), 35 families (family was defined as seeds sampled of individuals seed trees), 17–69 replicates, one plant per plot, and tree spacing of 6 × 1.5 m. At 18 months after planting, we measured the percent of family survival (SUR) and individual offspring height (*H*). The percent rate of SUR offspring per family was calculated as SUR = (*n*_surv_/*n*)100%, where *n*_surv_ is the number of surviving offspring per family, and *n* is the number of planted offspring per family. The individual offspring height was measured using a ruler (cm).

### 2.2 Microsatellite genotyping

DNA extraction from leaves of adult trees, juveniles, and germinated open‐pollinated seeds (offspring), amplification reactions, and microsatellite analysis procedures followed the methods described by Ciampi, Azevedo, Gaiotto, Ramos and Lovato (2008) and Moraes and Sebbenn (2011). We used six dinucleotide microsatellite loci (Hc14, Hc17, Hc33, Hc39, Hc40, and Hc49), which were previous tested for our data (Moraes et al., 2016), for Mendelian inheritance and genetic linkage analyses based on the genotypes of the mother and open‐pollinated seeds, and for genotypic linkage disequilibrium based on the genotypes of adults and juveniles. The six loci present Mendelian inheritance, an absence of genetic linkage, genotypic linkage disequilibrium and high levels of polymorphism (Moraes et al., 2016).

### 2.3 Analysis of genetic diversity

The genetic diversity for adults, juveniles, and the offspring of each population was quantified by the total number of alleles across all loci (*k*), allelic richness (*R*), and observed (*H*_o_) and expected (*H*_e_) heterozygosity. We estimated the fixation index (*F*), and its statistical significance was calculated by the permutation of alleles among individuals (600 randomizations). These analyses were carried out using FSTAT software (Goudet, 2002). However, as the *F* estimated for offspring can be biased due to the overestimation of gene frequencies of maternal alleles (each plant within a family receives at least one maternal allele), this index was estimated as described in Manoel et al. (2015). To test if the estimated indices were significantly different between samples, we used the unpaired *t* test.

### 2.4 Analysis of spatial genetic structure and effective population size

The analysis of SGS was carried out for adults and juveniles based on the estimate of the coancestry coefficient (*θ*_*xy*_), as described in Loiselle, Sork, Nason and Graham (1995), using SPAGEDI (Hardy & Vekemans, 2002). To compare the SGS of PA and PF adults and PF juveniles, we arbitrarily chose to use the same 11 distance classes between samples (25–1,000 m). We obtained the statistical significance of *θ*_*xy*_ by comparing the confidence interval limits at a 95% probability of the average estimate *θ*_*xy*_ for each distance class, as calculated by the permutation of individuals among distance classes. To compare the extent of SGS between PA and PF adults and PF juveniles, the *S*_p_ statistic (Vekemans & Hardy, 2004) was calculated. To test for the statistical significance of SGS, the spatial positions of the individuals were permuted 1,000 times. The group coancestry (Θ) for the adults and juveniles was estimated following Lindgren and Mullin (1998), and effective population size was calculated as described in Sebbenn et al. (2011).

### 2.5 Parentage analysis

Pollen flow, seed flow, dispersal distance, the combined exclusion probability of the first (*P*_1_) and second (*P*_2_) parent, and the combined exclusion probability of identity (*Q*_*i*_) were calculated using CERVUS 3.0 software (Kalinowski, Taper & Marshall, 2007). The cryptic pollen and seed flow (*C*_gf_) values were estimated as described in Dow and Ashley (1996). Parentage (maternity and paternity) analyses were based on the single exclusion method, assuming no mismatching among the offspring–(or juvenile)–mother–father trio. Due to the fact that our parentage (maternity and paternity) analyses were based on only six microsatellite loci, and the number of candidate putative parents was high (359 + 111 = 470 adults), which may have resulted in a high probability of cryptic gene flow (see Section 3), we chose to be conservative, and we only accepted the positive assignment of seeds and juveniles to parents, assuming no mismatching among the offspring–(or juvenile)–mother–father trio. Offspring and juveniles that were not assigned any parent in the populations were determined to originate from gene immigration. Individuals that received the same parent individual for both the maternal and paternal assignment were identified as resulting from self‐fertilization. We estimated the effective (for offspring) or realized (for juveniles) selfing rate (*s*) and the outcrossing rate from nonrelated individuals (*t*_u_) and from related parents (*t*_r_) based on the genotyped offspring of the provenance and progeny test. The selfing rate (*s*) was estimated as s = *n*_s_/*n*_1_, where *n*_s_ is the number of individuals originating from self‐fertilization, and *n*_1_ is the total number of sampled seeds or juveniles of each population. The outcrossing rate was estimated as *t *= 1 − *s*. Outcrossed individuals were classified as originating from mating among nonrelated (*t*_u_) or related (*t*_r_) parents. Offspring and juveniles from *t*_r_ were determined using the estimate of the coancestry coefficient (*θ*_*xy*_) between assigned parents in SPAGEDI 1.3 (Hardy & Vekemans, 2002). Following Ismail et al. (2014), if *θ*_*xy*_ ≥ 0.1 between the assigned parents, we assumed that the offspring or juvenile was inbred due to mating between related parents. The *t*_u_ value was calculated as *t*_u_ = *n*_u_/*n*_1_, and for related parents, *t*_r_ = *n*_r_/*n*_1_, where *n*_u_ and *n*_r_ are the number of individuals originating from nonrelated parents and related parents, respectively. To confirm the results of the parentage analysis, we estimated the mean *θ*_*xy*_ between offspring or juveniles and their assigned parents. We estimated the mean, standard deviation, and minimum and maximum for *θ*_*xy*_ within families. The spatial positions of adults and juveniles (*x* and *y* coordinates) were used to estimate the mean, standard deviation (*SD*), median, minimum and maximum pollen and seed dispersal distances, based on the Euclidian distance between two points. To investigate if reproductive success was a function of the distance between trees, we compared the frequency distribution of pollen dispersal to the frequency distribution of the distance between all trees of both populations using the Kolmogorov–Smirnov test. The effective pollination neighbor area (*A*_ep_) was calculated as described in Levin (1988), and the effective radius of pollen dispersal was based on Austerlitz and Smouse (2002). To investigate male and female fertility and whether trees with the greatest dbh produced more offspring and juveniles as pollen donor parents (male fertility) or maternal parents (female fertility), we used the Spearman correlation coefficient (*ρ*).

### 2.6 Analysis of mating system by MLTR

We assessed the mating system at the population and individual levels based on the Expectation–Maximization numerical method (EM) using MLTR 3.1 software (Ritland, 2002). The estimated indices were the maternal fixation index (*F*_m_); multilocus (*t*_m_) and single‐locus (*t*_s_) outcrossing rates; mating among related individuals (*t*_m_ − *t*_s_); correlation of selfing (*r*_s_); correlation of selfing among loci (*r*_s(l)_); paternity correlation within and among fruits (*r*_p_), within fruits (*r*_p(w)_), and among fruits (*r*_p(a)_); and gene frequencies of pollen and ovules. The standard deviations of these indices were estimated by 1,000 bootstraps, using individuals within families as candidate he units of resampling. The effective number of pollen donors within and among fruits (*N*_ep_ = 1/*r*_p_), within fruits (*N*_ep(w)_ = 1/*r*_p(w)_), and among fruits (*N*_ep(a)_ = 1/*r*_p(a)_) was calculated following Ritland (1989). The mean coancestry coefficient (Θ) and variance effective size (*N*_e_) within families was estimated based on Sebbenn (2006), and the number of seed trees for seed collection (*m*) was calculated to retain an effective reference size of 150 in the total sampled progeny array (Sebbenn, 2006). The 95% confidence interval of the indices was estimated as described in Wadt et al. (2015). To determine the associations among the sample size (*n*), *R*,* H*_o_, *F*,* s* + (*t*_m_ − *t*_s_), *N*_ep_, and *N*_e_ within families, we used Spearman's rank correlation coefficient (*ρ*).

### 2.7 Analysis of inbreeding depression

Inbreeding depression for offspring of the provenance‐progeny test was assessed in terms of selfing (*δ*_s_) and mating among relatives (*δ*_r_) as:

$$\delta_{s}=1-\left(\frac{x_{s}}{x_{t_{u}}}\right)$$

and

$$\delta_{r}=1-\left(\frac{x_{t_{r}}}{x_{t_{u}}}\right),$$

where $x_{t_{u}}$, $x_{t_{r}}$, and *x*_s_ are the means of the traits (survival and height) for offspring originating from outcrossing between unrelated individuals, outcrossing between related individuals, and self‐fertilization, respectively, based on paternity analysis and the coancestry coefficient between the assigned parents.

## 3 RESULTS

### 3.1 Genetic diversity

For all adults, juveniles, and offspring of both populations (1,565), we found 99 alleles across the six loci (Table 1). Based on an unpaired *t* test, the mean allelic richness (*R*) was significantly higher in the PA adults and offspring than the PF adults, offspring, and juveniles; the observed heterozygosity (*H*_o_) was significantly higher in adults than offspring for both populations; and, for the PF population, the expected heterozygosity (*H*_e_) was significantly higher in adults than in offspring. For both populations, the fixation index (*F*) was significantly lower than zero in adults and juveniles, significantly higher than zero in offspring, and significantly lower in adults than offspring.

**Table 1:** Genetic diversity and fixation index (*F*) for adults, offspring, and juveniles in a pasture (PA) and a forest fragment (PF)

Sample	*n*	*k*	*R* (*SD*)	*H*_o_ (*SD*)	*H*_e_ (*SD*)	*F* (*SD*)	Θ	*N*_e_	*N*_e_/*n*
PA: adults	359	92	14.6 (2.8)a	0.96 (0.02)a	0.87 (0.04)a	−0.11 (0.05)a^a^	0.003171	158	0.44
PA: offspring	457	81	11.5 (2.4)a	0.48 (0.07)b	0.84 (0.03)a	0.43 (0.08)b^a^	—	—	—
PF: adults	111	53	8.8 (3.5)b	0.93 (0.04)a	0.83 (0.05)a	−0.13 (0.09)a^a^	0.007127	70	0.63
PF: offspring	419	53	8.2 (2.8)b	0.51 (0.20)b	0.78 (0.07)b	0.35 (0.23)b^a^	—	—	—
PF: juveniles	219	54	8.8 (3.1)b	0.90 (0.04)a	0.82 (0.06)a	−0.11 (0.09)a^a^	0.005384	93	0.42
Total	1,565	99	—	0.69 (0.08)	0.83 (0.04)	—	—	—	—

*Notes*

Different letters mean significant differences at the 5% probability level of the unpaired *t* test.

*H*_e_ is the expected heterozygosity; *H*_o_ is the observed heterozygosity; *k* is the total number of alleles; *n* is the sample size; *N*_e_ is the effective population size; *R* is the allelic richness for 111 individuals genotyped for six loci; *SD* is the standard deviation; Θ is the group coancestry coefficient.

a *p* < 0.05.

### 3.2 Spatial genetic structure and effective population size

**Figure 2:**
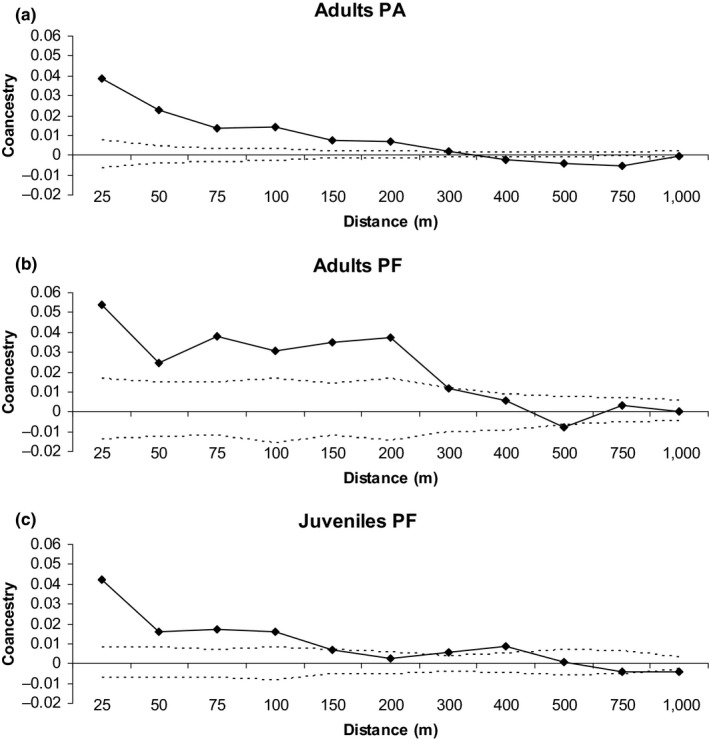
Spatial genetic structure in the adults of PA (a) and PF (b) and the juveniles of PF (c). The continuous line represents the average estimated coancestry coefficient described in Loiselle et al. (1995), and the dashed lines represent the confidence interval at the 95% probability of the hypothesis of no spatial genetic structure (H0: *θ*_*xy*_ = 0)

For the adults of both populations, the spatial distribution of genotypes (SGS) was significantly structured up to 250 m, and for PF juveniles, it was significantly structured up to 125 m (Figure 2). The strength of SGS was similar among the adults of PA (*S*_p_ = 0.014) and PF (*S*_p_ = 0.009) and the juveniles of PF (*S*_p_ = 0.015). The group coancestry (Θ) for adults and juveniles was low (Θ < 0.003), indicating that during random mating, a low level of inbreeding should be expected (<1%). The effective population size (*N*_e_) was lower than the sample size of adults and juveniles (*N*_e_/*n* < 1), especially for PF juveniles and PA adults.

### 3.3 Parentage analysis

**Figure 3:**
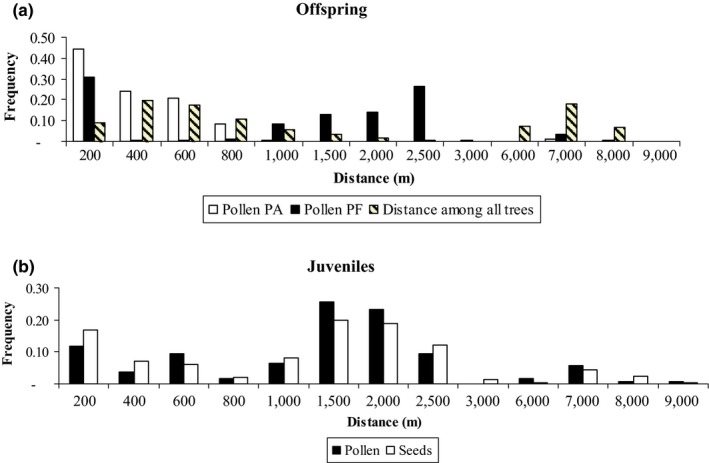
Effective pollen dispersal distance for the PA and PF offspring and distance between trees in the PF and PA populations (a), and the distance of realized pollen and seed dispersal in PF juveniles (b)

**Figure 4:**
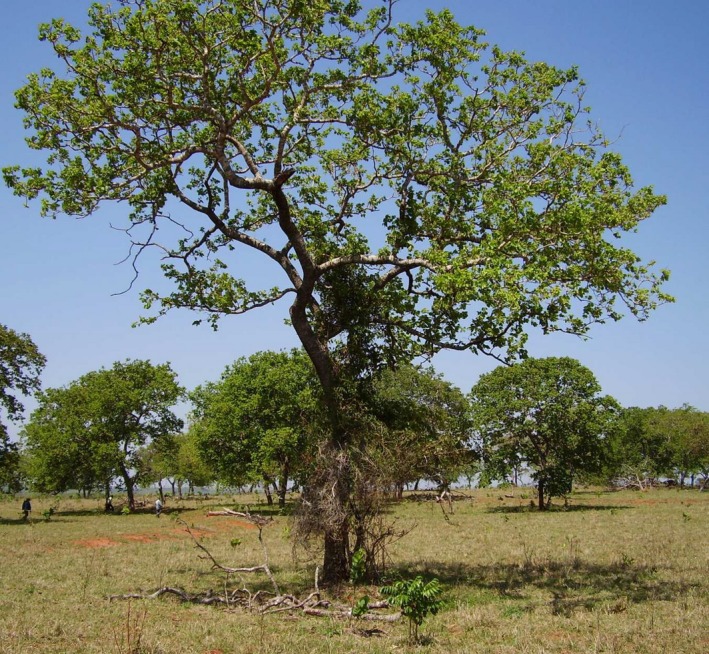
Area of seed collection for *Hymenaea stigonocarpa* trees in the pasture (PA)

For all 470 adults from both populations, the combined exclusion probabilities of the first (*P*_1_ = 0.9950) and second (*P*_2_ = 0.9997) parent were very high, indicating a high probability of cryptic pollen and seed flow for juveniles (0.905 = 1 − 0.9950^470^) and a low probability of these parameters for offspring (0.113 = 1 − 0.9997^470^). Thus, the observed levels of both pollen and seed flow may be biased for juveniles. The combined nonexclusion probability of identity (*Q*_*i*_) was low (8.06^−07^), indicating that all adults present different genotypes, which is necessary for the assignment of parents in parentage analysis. A pollen donor was found for 80.1% of the offspring in PA and 78.8% of the offspring in PF, indicating a pollen immigration of 19.9% and 21.2% in PA and PF, respectively, from trees located outside of both sampled populations (Table 2). For PA, 79.4% originated from within the population, and 20.6% originated from outside the PA, with 0.7% originating from PF. In PF, 76.1% of the pollen came from within the population, and 23.9% came from outside PF, with 2.6% originating from PA. For PF juveniles, two putative parents were found for 85.8% of the genotypes, with 14.2% not assigned two parents (realized pollen immigration); 76.1% of the parent pairs were assigned from within PF, and 23.9% were from outside PF, with 2.6% originating from PA. Assuming that a single assigned parent represents the mother, at least one putative mother was assigned for 96.8% of the juveniles, with 3.2% not from within the two populations (realized seed immigration), 83.6% originating from mothers located within PF and 16.4% from mothers outside of PF, with 13.2% of mother trees from the PA population. The mean pairwise coancestry between juveniles and the first (assumed as the mother: *θ*_1_ = 0.19) and second (assumed as the father: *θ*_2_ = 0.19) parent were lower than expected (0.25). The mean pairwise coancestry between offspring and the mother (PA: *θ*_1_ = 0.42; PF: *θ*_1_ = 0.32) and between offspring and the father in PF (*θ*_2_ = 0.29) were higher than expected (0.25), with the exception of *θ*_2_ in PA (0.13). The total mean pairwise coancestry within families (*θ*_w_) in PA was 0.398, ranging from 0.276 to 0.630 among families, and in PF, it was 0.306, ranging from 0.139 to 0.529 among families (Supporting Information Table S1). For offspring assigned a father, the mean and maximum pollen dispersal distances were lower in PA (365/6,899 m) than in PF (1,401/7,746 m); however, the median distances (PA = 267 m, PF = 1,388 m) were lower than the means in both populations, indicating a pollen dispersal pattern of isolation by distance (IBD) (Figure 3). The effective pollination neighbor area (*A*_ep_) and radius (*r*_ep_) were also lower in PA (206 ha/809 m) than in PF (1,233 ha/1,981 m). The results from the Kolmogorov–Smirnov test indicate that the frequency distribution of pollen dispersal and distances among all trees (Figure 3a) were statistically (*p* < 0.001) different in both PA (*D* = 0.429) and PF (*D* = 0.281). Thus, the spatial distance between trees does not explain the pollen dispersal patterns in these populations. The pollen dispersal pattern was also significantly different between PA and PF (*D* = 0.647, *p* < 0.001). For juveniles with two assigned parents, the mean distance of pollen dispersal (1,640 m) was higher than the median distance (1,339 m), also suggesting an IBD pattern of realized pollen dispersal. For the juveniles with at least one parent found within the populations, the mean minimum and maximum seed dispersal distances (808/2,237 m) were higher than the median distances (552/1,678 m), again indicating an IBD pattern. We found no association between dbh and male fertility in the PA population (*ρ *= 0.018, *p* < 0.841). However, a significantly positive association was detected for offspring (*ρ *= 0.247, *p* < 0.027) and for male and female fertility in juveniles (*ρ *= 0.275, *p* < 0.010) of the PF population. These results indicate that in PF, trees with a greater dbh produce a greater number of offspring and juveniles than trees with a lower dbh (Figure 4).

**Table 2:** Results of CERVUS parentage analysis for PA and PF offspring (pollen) and PF juveniles (pollen and seeds)

	Offspring (PA and PF): pollen		Juveniles (PF)	
	PA	PF	Pollen	Seeds
Parentage assignments				
Sample size: *n*	457	419	219	219
Genotypes assigned for at least one parent (%)	366 (80.1)	330 (78.8)	188 (85.8)	212 (96.8)
Genotypes not assigned within both PA and PF (%)	91 (19.9)	89 (21.2)	31 (14.2)	7 (3.2)
Genotypes assigned within population (%)	363 (79.4)	319 (76.1)	172 (78.5)	183 (83.6)
Genotypes not assigned within PA or PF (%)	94 (20.6)	100 (23.9)	47 (21.5)	36 (16.4)
Genotypes assigned between PF and PA (%)	3 (0.7)	11 (2.6)	16 (7.3)	29 (13.2)
Coancestry: first parent: *θ*_1_ ± *SD*	0.42 ± 0.12	0.32 ± 0.15	0.19 ± 0.17	—
Coancestry: second parent: *θ*_2_ ± *SD*	0.13 ± 0.11	0.29 ± 013	0.19 ± 0.18	—
Dispersal distance				
Mean dispersal distance: *D *± *σ* (m)	365 ± 572	1,401 ± 1,401	1,640 ± 1,645	808 ± 825
Median dispersal distance (m)	267	1,388	1,339	552
Minimum/maximum dispersal distance (m)	13/6,899	1/7,746	8/8,163	5/8,091
Effective pollination neighbor area: *A*_ep_ (ha)	206	1,233	270	—
Effective pollen dispersal distance radius: *r*_ep_ (m)	809	1,981	2,326	—
Mating system				
Selfing: *s* (%)	112 (24.5)	57 (13.6)	0 (0)	—
Outcrossing: *t* = *t*_u_ + *t*_r_ (%)	345 (75.5)	362 (86.4)	219 (100)	—
Mating among nonrelatives: *t*_u_ (%)	199 (43.6)	260 (62.1)	210 (95.9)	—
Mating among relatives: *t*_r_ (%)	146 (31.9)	102 (24.3)	9 (4.1)	—
Coancestry between relative parents: *θ*_r_ ± *SD*	0.21 ± 0.07	0.18 ± 0.07	0.17 ± 0.05	—
Mean distance for *t*_r_: *D*_r_ ± *σ* (m)	272 ± 195	1,121 ± 893	310 ± 468	—
Fixation index				
Fixation index for selfed: *F*_s_ ± *SD*	0.65 ± 0.21	0.61 ± 0.26	—	—
Fixation index for *t*_r_: *F*_r_ ± *SD*	0.36 ± 0.19	0.36 ± 0.17	0.09 ± 0.10	—

*Notes*

The maximum mean seed dispersal distance was 2,237 ± 1,982 m, and the median distance was 1,687 m.

*SD* is the standard deviation; *σ* is the square root of the axial variance.

Among the offspring, 24.5% from PA and 13.6% from PF were found to be the result of selfing (*s*), indicating an outcrossing rate (*t*) of 75.5 and 86.4%, respectively (Table 2). Outcrossing among nonrelatives (*t*_u_) was higher (PA = 43.5%, PF = 62.1%) than outcrossing among related individuals, *t*_r_ (PA = 31.9%, PF = 24.3%). The mean coancestry among related parents (*θ*_r_) in PA (0.21) was higher than that in PF (0.18), but the mean distance (*D*_r_) was lower in PA (272 m) than in PF (1,121 m). The mean fixation index for selfed offspring (*F*_s_) was higher than expected (0.5), and the mean fixation index for offspring produced through *t*_r_ (*F*_r_) in PA (0.36) and PF (0.36) was higher than *θ*_r_. All juveniles were the result of outcrossing, with 4.1% from *t*_r_, a mean distance between related parents (*D*_r_) of 310 m, and a *F*_r_ of 0.08, which was lower than *θ*_r_ (0.17).

### 3.4 Mixed mating system hierarchy within and among fruits by MLTR

The mean population fixation index of seed trees (*F*_m_) was not significantly different from zero (Table 3), but the individual *F*_m_ was lower than *F*_o_ for 51.7% of the families. These results suggest selection against inbred individuals between seed and adult stages (Supporting Information Table S2). The multilocus outcrossing rate (*t*_m_) was significantly lower than the unity (PA = 0.77, PF = 0.81) and variable among seed trees (0.53–1.0), indicating that some offspring were produced through self‐fertilization. The selfing correlation (*r*_s_) was significantly higher than zero, confirming the individual variation for *t*_m_. Mating among related individuals (*t*_m_ − *t*_s_) was significantly higher than zero in 19 families of PA and 12 families of PF. The rate of mating among relatives was also significantly higher in PA than in PF. The correlation of selfing among loci (*r*_s(l)_) was low (<0.12), indicating that inbreeding within the populations was mainly the result of selfing. The paternity correlations within and among (*r*_p_), within (*r*_p(w)_), and among (*r*_p(a)_) fruits were not significantly different between populations, indicating that seed trees were fertilized by a limited number of pollen donors (maximum *N*_ep_ = 2). However, all estimates of the paternity correlation and effective number of pollen donors were variable among seed trees, with *r*_p(w)_ higher than *r*_p(a)_ in 92% of the families, suggesting a higher probability of full‐sibs occurring within fruits than among fruits. The coancestry (Θ) and effective size (*N*_e_) within families of both populations were similar to those expected for full‐sibs (Θ = 0.25, *N*_e_ = 2). The number of seed trees (*m*) for seed collection was not significantly different between PA (80) and PF (75). The individual genetic diversity indices *R* and *H*_o_ were also variable among families (Supporting Information Table S2). The Spearman rank correlation (*ρ*) (Supporting Information Table S3) was positively significant between the indices *N*_ep_ vs. *R*,* N*_e_ vs. *R*, and *N*_e_ vs. *H*_o_. These results indicate that the effective number of pollen donors (*N*_ep_) increases allelic richness (*R*), and high *R* and observed heterozygosity (*H*_o_) values increase the effective size within families (*N*_e_). Negative and significant associations were detected between the sum of the rates of selfing and mating among relatives (*s* + (*t*_m_ − *t*_s_)) compared to *R*,* H*_o_, *N*_ep_, and *N*_e_, as well as the fixation index (*F*) versus *H*_o_ within families. Thus, high levels of selfing and mating among related individuals decrease the allelic diversity, heterozygosity, effective number of pollen donors and effective size, and inbreeding decreases heterozygosity within families. The Spearman ranking correlation coefficient was also significantly positive (*ρ* = 0.608, *p* = 0.001) between the estimated mean coancestry within families from the mating system indices (Θ) and the estimated mean pairwise coancestry within families (*θ*_w_) based on the methods of Loiselle et al. (1995).

**Table 3:** Mean and 95% confidence interval (95% CI) results for the mating system indices in the PA and PF populations

	PA (95% CI)	PF (95% CI)
Fixation index of seed trees: *F*_m_	0.09 (0.00–0.14)	−0.02 (−0.20 to −0.03)
Multilocus outcrossing rate: *t*_m_	0.77 (0.71–0.84)	0.81 (0.74–0.89)
Mating among relatives: *t*_m_ − *t*_s_	0.46 (0.43–0.50)	0.30 (0.26–0.30)
Selfing correlation: *r*_s_	0.08 (0.04–0.14)	0.11 (0.05–0.16)
Selfing correlation among loci: *r*_s(l)_	0.11 (0.05–0.17)	0.16 (0.13–0.42)
Paternity correlation: *r*_p_	0.51 (0.34–0.59)	0.65 (0.40–0.74)
Number of pollen donor: *N*_ep_	2.0 (1.9–3.0)	1.5 (1.2–2.5)
Coancestry within family: Θ	0.252 (0.200–0.286)	0.235 (0.197–0.256)
Effective size within family: *N*_e_	1.87 (1.66–2.31)	2.01 (1.83–2.39)
Seed trees for seed collection: *m*	80 (65–91)	75 (63–82)
Among and within fruits		
Paternity correlation within: *r*_p(w)_	0.51 (0.35–0.59)	0.63 (0.37–0.73)
Paternity correlation among: *r*_p(a)_	0.51 (0.33–0.60)	0.67 (0.42–0.76)
Number of pollen donors: *N*_ep(w)_	2.0 (1.7–2.9)	1.6 (1.4–2.7)
Number of pollen donors: *N*_ep(a)_	2.0 (1.7–3.0)	1.5 (1.3–2.4)

### 3.5 Inbreeding depression

The rate of survival (SUR) of the offspring at 18 months after establishing the provenance and progeny test (Table 4) was lower in PA (54.5%) than in PF (63.7%). The survival rate of offspring produced by mating among unrelated (*t*_u_) individuals (49%–66.7%) was higher than that of offspring produced by selfed (*s*) individuals (12%–19.3%) and mating among related (*t*_r_) individuals (21.3%–31.7%). The mean height (*H*) was also greater for offspring from *t*_u_ than for offspring from *t*_r_ and *s*. The inbreeding depression (ID) for SUR and plant height (*H*) was greater for offspring originating from *s* than those originating from *t*_r_.

**Table 4:** Survival (SUR), mean height (*H*), and inbreeding depression for selfing (*δ*_s_) and mating among related trees (*δ*_r_) at 18 months for offspring of the PA and PF populations

	PA		PF	
	SUR	*H*	SUR	*H*
Sample size: *n*	457	457	419	419
Total survival and mean *H*	249 (54.5%)	28.7 cm	267 (63.7%)	35.3 cm
Selfing: *s*	48 (19.3%)	25.7 cm	32 (12.0%)	27.1 cm
Outcrossing: *t* = *t*_u_+*t*_r_	201 (80.7%)	29.7 cm	235 (88.0%)	36.6 cm
Mating among nonrelated trees: *t*_u_	122 (49.0%)	31.8 cm	178 (66.7%)	39.1 cm
Mating among related trees: *t*_r_	79 (31.7%)	26.8 cm	57 (21.3%)	30.2 cm
Inbreeding depression: *δ*_s_	60.7%	19.2%	82.0%	30.7%
Inbreeding depression: *δ*_r_	35.2%	15.7%	68.0%	22.8%

## 4 DISCUSSION

Our study compares adult *H. stigonocarpa* individuals that are remnants of preforest fragmentation phases with juveniles and open‐pollinated offspring from the postfragmentation period. A previous study, which investigated the mating system and effective pollen dispersal of a small fragmented population of *H. stigonocarpa* and six isolated trees in a pasture from the same savannah region of the present study, found that pollen dispersal could cross long distances and the rate of self‐fertilization was higher in isolated trees in the pasture than in trees located in the forest fragment (Moraes & Sebbenn, 2011). However, this previous studied used limited sample sizes of adult trees and seeds sampled from the forest fragment (28 adults and 137 seeds) and isolated trees and seeds sampled from the pasture (six adults and 34 seeds); furthermore, the realized pollen and seed dispersal in juvenile trees were never investigated. Due to that, we carried out the present study using higher sample sizes of adults and seeds from a forest fragment (111 adults and 450 seeds) and isolated trees and seeds from a pasture (359 adults and 600 seeds), and we also sampled juvenile trees (219) within the forest fragment, aiming to compare our results with the previous results and investigate the realized pollen and seed immigration, dispersal distance and patterns with a high sample size of reproductive trees and open‐pollinated seeds in the different environments. We studied populations in two contrasting environments approximately 5 km apart, one in a pasture (PA) and the other in a forest fragment (PF). Our results reveal important information about the effects of evolutionary forces and processes on subsequent tree generations in this landscape, such as long‐distance pollen and seed dispersal, gene dispersal patterns of isolation by distance, genetic drift within the populations, and inbreeding depression in terms of survival and plant growth. These results have important practical applications for genetic conservation, tree breeding, and environmental reforestation plans.

### 4.1 Genetic diversity

The mean allelic richness (*R*) was higher in adults and offspring from PA than those from PF. Adult individuals in these populations are remnants of prefragmentation phases (which occurred approximately 50 years ago), which may explain the differences in the genetic diversity levels between the populations. The PA adults, although in a highly modified habitat, retain levels of genetic diversity from the prefragmentation period, which may have been higher than that of the near‐neighbor PF population. This result indicates that the PA population is important for the maintenance of the species in the landscape.

The mean observed heterozygosity (*H*_o_) was higher and the fixation index (*F*) and was lower in adults than in offspring of both populations. Mating patterns in the parental populations, such as self‐fertilization, mating among related individuals, and correlated mating, can explain the low genetic diversity and inbreeding in offspring. However, the absence of inbreeding in adults suggests selection against inbred individuals between the offspring and adult stages. Furthermore, the strong inbreeding depression detected for survival suggests that inbred offspring are not likely to survive to adulthood. Furthermore, the observed low *H*_o_ and high *F* levels in offspring likely change to higher *H*_o_ and lower *F* levels when these individuals reach the adult stage.

### 4.2 Spatial genetic structure and seed dispersal

Adults of both populations and PF juveniles present a SGS. Thus, there is a high probability that near‐neighbor adults located within 280 m (PA) or 350 m (PF) and PF juveniles within 125 m are related. SGS is mainly determined by short‐distance seed dispersal, although short‐distance pollen dispersal can also contribute to SGS (Collevatti, Lima, Soares & Telles, 2010; Hardy et al., 2006). For *H. stigonocarpa*, SGS is likely the result of short‐distance seed dispersal near the mother trees, with pollen dispersal contributing minimally to the creation of SGS. Seed dispersal reached long distances (up to 8,091 m), but the dispersal pattern was IBD, with a high frequency of juveniles established near parent trees, which can explain the SGS. The seed and pollen dispersal vectors of the species have the potential for long‐distance dispersal. *Hymenaea stigonocarpa* seeds are dispersed by large mammals (*Agoutis agoutis*) and birds (Ramos et al., 2009), and, as discussed above, pollen is dispersed by bats (Gribel & Lemos, 1999; Lacerda, Kanashiro & Sebbenn, 2008) that frequently travel more than 200 m between trees (Dunphy, Hamrick & Schwagerl, 2004). In the present study, the sampled populations are surrounded by sugar cane and *Eucalyptus* plantations, resulting in a limited presence of animals, such as large mammals, to disperse seeds. This lack of seed dispersers results in seedling establishment near the mother‐tree, thus increasing the probabilities of mating among relatives and inbreeding in subsequent generations. Our results confirm these expectations, as mating among related individuals (*t*_r_ and *t*_m_ − *t*_s_) was detected by the parentage analysis for seeds (*t*_r_: 24.3%–31.9%) and juveniles (*t*_r_ = 4.1%), by the MLTR mixed mating system analysis for both seeds (*t*_m_ − *t*_s_: 30%–46%) and by the inbreeding in seeds (0.35–0.43). However, the impact of seed dispersal on the genetic composition of tree populations is determined by the survival of seeds to reproductive age. In many cases, the establishment of seeds is density‐ and distance‐dependent and controlled by predation and environmental characteristics (Muller‐Landau, Wright, Calderón, Condit & Hubbell, 2008). Thus, the realized seed dispersal is associated not only with the type of dispersers but also with the location and degree of fragmentation of the environment, which can interfere with disperser behavior. In the present case study, the extensive level of anthropogenic disturbances in the region may favor the mobility of *H. stigonocarpa* seed dispersal vectors due to the presence of isolated trees in the pasture creating connectivity corridors with the forest fragment, as seen through seed immigration from PA into PF (13.2%) and from outside both populations (3.2%). In this context, it is important to conserve both populations to preserve the ecosystem dynamics and maintain gene immigration into PF.

The strength of SGS in PF is similar between adults (*S*_p_ = 0.009) and juveniles (*S*_p_ = 0.015). The *S*_p_ values for PF adults (PA: *S*_p_ = 0.014; PF: *S*_p_ = 0.009) and PF juveniles (*S*_p_ = 0.015) were similar to those detected for plants with seeds dispersed by animals (*S*_p_ = 0.0088, Vekemans & Hardy, 2004) and pollen dispersed by animals (*S*_p_ = 0.0171, Vekemans & Hardy, 2004), for trees with seeds dispersed by birds, bats, and monkeys (*S*_p_ = 0.009; Dick, Hardy, Jones & Petit, 2008), bat‐pollinated and animal seed‐dispersed trees, such as *Caryocar brasiliense* (*S*_p_ = 0.0116, Collevatti et al., 2010), and trees with pollen dispersed by animals (*S*_p_ = 0.0171, Vekemans & Hardy, 2004). Our *S*_*p*_ values are lower than those reported for other tropical trees, such as: *Swartzia glazioviana*, which is insect‐pollinated, with seeds primarily dispersed by barochory and vegetative propagation (*S*_p_ = 0.028–0.063, Spoladore, Mansano, Lemes, Freitas & Sebbenn, 2017); *Theobroma cacao*, which is insect‐pollinated, with seeds dispersed by small animals and birds and vegetative propagation (*S*_p_ = 0.0209, Silva, Albuquerque, Ervedosa, Figueira & Sebbenn, 2011); and *Copaifera langsdorffii*, which is insect‐pollinated, with seeds dispersed by monkeys and birds (*S*_p_ = 0.0246–0.0259, Sebbenn et al., 2011). However, our results are higher than those found for the tropical trees *Himatanthus drasticus* (*S*_p_ = 0.0044–0.0448, Baldauf et al., 2014) and *Tabebuia alba* (*S*_p_ = 0.0061, Braga & Collevatti, 2011), both which are insect‐pollinated, with seeds dispersed by the wind. The results from previous studies and those reported herein suggest that species with pollen and seeds dispersed by animals present a lower SGS than insect‐pollinated trees with seeds dispersed by barochory but present a greater SGS than insect‐pollinated trees with seeds dispersed by the wind.

### 4.3 Effective population size

The effective population size (*N*_e_) was lower than the sample size (*n*) for the adults and juveniles (*N*_e_/*n *<* *1) of the populations due to the occurrence of SGS. Related individuals present identical‐by‐descent alleles, which decreases the effective population size (*N*_e_) in populations. The effective population size (*N*_e_) is a key variable for conservation genetics, as populations with a low effective population size (*N*_e_) can lose genetic diversity and increase inbreeding, resulting in inbreeding depression and reduced population fitness (Kalinowski & Waples, 2002). Therefore, for in situ conservation, a minimum effective population size (*N*_e_) of 70 has recently been suggested for random mating populations to avoid inbreeding depression (Caballero, Bravo & Wang, 2016). Although they occur in a significantly fragmented landscape, the adults and juveniles of our studied populations presented effective population size (*N*_e_) values (70–158) greater than 70, indicating that both the PA and PF populations have an effective population size (*N*_e_) sufficient for in situ conservation. However, as the populations present deviation of random mating due to selfing, mating among related trees and correlated mating, we must consider using a reference effective population size of 150, as suggested by Sebbenn (2006), for in situ conservation. This indicates that the effective population size (*N*_e_) of the PF population must be increased by, for example, the plantation of 80 individuals that are neither inbred nor related.

### 4.4 Gene flow

Our results show that the two studied populations are not genetically isolated due to pollen and seed flow between the PA and PF populations and from individuals not included in the analysis located outside of the study area. Pollen immigration levels in PA (20.6%) and PF (21.5%–23.9%) were similar, but pollen flow into PF from PA was greater (2.6%–7.3%) than pollen flow from PF into PA (0.7%), suggesting that bats tend to move from pastures to forests. Furthermore, in PF, the realized pollen flow (*m*_p_) was 1.3 times higher (*m*_p_/*m*_s_ = 21.5%/16.4%) than the seed flow (*m*_s_), with pollen immigration mainly coming from outside both populations (*m*_p_/*m*_s_ = 14.2%/3.2% = 4.4) and seed immigration mostly coming from PA (*m*_p_/*m*_s_ = 7.3%/13.2% = 0.55). Although the inbreeding depression and stochastic factors, such as random mortality, predation, and diseases, may change the effective to realized pollen and seed dispersal stages, these results suggest that bats are more efficient gene dispersers than large mammals and birds. To understand the pollen flow of a species, we must first consider the behavior of its pollinators. Bats need to consume 1 mg of sugar or approximately 5 μl of nectar, and flower nectar has a sugar concentration of 20% (Nassar, Ramirez & Linares, 1997). In the savannah region of Brazil, Bobrowiec and Oliveira (2012) studied the relationship between pollination and plants for four species pollinated by bats, including *H. stigonocarpa*. This species showed higher levels of nectar production (430 μl/hr) and nectar sugar concentration (701 mg) and fewer flowers visited per tree (five flowers) than the other species included in the study. Another important factor is the morphology of the plants’ pollen grains. The *H. stigonocarpa* pollen grains are large, dense, and oval shaped, and they easily adhere to animals; therefore, the mixing of pollen when bats feed is common (Stroo, 2000). These considerations may explain the fact that both populations present high rates of pollen immigration and selfing and a low effective number of pollen donors between and within fruits.

Our results also show that the realized pollen flow was greater than the effective pollen flow. However, because juveniles differ in height, the realized pollen flow likely represented more than one reproductive event, while the effective pollen flow represented a single reproductive event (open‐pollinated seeds). Gene flow depends on many factors, such as flowering phenology and pollinator behavior that can vary between reproductive events. Thus, these factors may explain the observed differences between realized and effective pollen flow.

### 4.5 Pollen dispersal distance

The pollen dispersal across the studied landscape reached long distances (maximum of 8,163 m). Nevertheless, this maximum distance may be underestimated due to pollen immigration from outside the study area. Bats are capable of traveling long distances, as previously reported for *H. stigonocarpa* (7,353 m; Moraes & Sebbenn, 2011) and other bat‐pollinated trees, with a distance of 1,943 m reported for *Hymenaea courbaril* (Lacerda et al., 2008) and a distance of 18 km reported for *Ceiba pentandra* (Gribel & Lemos, 1999). However, the observed pollen dispersal pattern for *H. stigonocarpa* was isolation by distance, with most pollination events occurring at distances less than 2,000 m; in PA, 89.4% occurred within 600 m, and in PF, 68.9% occurred within 2,000 m. We can attribute this pattern to the fact that bats feed less than two minutes per tree and often travel more than 200 m between trees (Dunphy et al., 2004). Although bats can travel long distances, they tend to forage at high frequencies between near‐neighbor trees. The curve of the pollen dispersal distance pattern was also different between PA and PF, which can be explained by the distribution of trees across the landscape or the tree population density, with the trees in PA having a lower range of distance (1–1,528 m) and population density (3.29 trees/ha) than the trees in PF (3–2,727 m; 0.17 trees/ha). The low population density, associated with other factors, such as variations in flower phonology, may increase the pollen dispersal distance since pollinator vectors must fly longer distances to collect nectar than they do in regions with higher tree population densities (Degen & Sebbenn, 2014; Dick et al., 2008; Tambarussi, Boshier, Vencovsky, Freitas & Sebbenn, 2015).

The effective pollination neighbor area (*A*_ep_) represents a circular area around a plant in which 63% of mating events are expected to occur (Levin, 1988). Our results indicate that the *A*_ep_ values (PA = 206 ha; PF = 1,233 ha) are larger than the area of the studied populations (PA = 109.12 ha; PF = 665.7 ha), and the effective pollination neighborhood radius (*r*_ep_) is lower in PA (809 m) than in PF (1,981 m). The higher effective pollination neighbor area (*A*_ep_) than the population area is due to pollen flow, and the lower effective pollination neighbor area (*A*_ep_) and effective pollination neighborhood radius (*r*_ep_) in PA than in PF are the results of the higher population density in PA, which is associated with an IBD pollen dispersal pattern. Another factor contributing to the high effective pollination neighbor area (*A*_ep_) is that *H. stigonocarpa* has a high level of nectar production per flower; therefore, bats, as the pollinator vector, only need to visit a few flowers per individual before moving on to the next tree, resulting in a wide pollen foraging area. In general, these effective pollination neighbor area (*A*_ep_) results are higher than the results reported for other tropical trees, such as the moth‐pollinated *Cordia alliodora* (24.9 ha, Boshier, Chase & Bawa, 1995), the bee‐pollinated *Carapa guianensis* (6.36 ha, Cloutier, Kanashiro, Ciampi & Schoen, 2007), *Cariniana legalis* (23–37 ha, Tambarussi et al., 2015), *Genipa americana* (5.1–11.2 ha, Manoel et al., 2017) and *Swietenia humilis* (209 ha, White, Boshier & Powell, 2002), and the bat‐pollinated *Caryocar brasiliense* (5.4 ha, Collevatti et al., 2010) and *H. courbaril* (169.9 ha, Lacerda et al., 2008); however, the results are lower than those found for wasp‐pollinated trees, such as *Ficus obtusifolia* (10,780 ha), *Ficus dugandii* (63,180 ha), and *Ficus popenoei* (29,480 ha, Nason & Hamrick, 1997). The larger effective pollination neighbor area (*A*_ep_) detected here for *H. stigonocarpa* than in other studies for *C. alliodora*,* C. legalis*,* C. guianensis*,* G. americana*,* S. humilis*,* C. brasiliense,* and *H. courbaril* can mainly be explained by the combination of the ability of bats to fly long distances and the large area studied here (2,523 ha). Pollen dispersal studies covering large areas may permit the detection of pollen dispersal events occurring between very distant trees. This can also explain why the effective pollination neighbor area (*A*_ep_) in this study was lower than those reported for *F. dugandii*,* F. obtusifolia*, and *F. popenoei*, which were investigated in studies with greater areas (>15,000 ha, Nason & Hamrick, 1997).

### 4.6 Parent fertility success

Female and male fertility in PF increased with tree dbh. Our results show that in PF, offspring, and juveniles were fathered by pollen donor trees with a high dbh, and juveniles were produced from mother trees with a high dbh. Thus, the observed IBD effective pollen dispersal and realized pollen and seed dispersal were determined in part by the size of the trees. Male fertility successes for trees with high dbh values and IBD pollen dispersal patterns have also been reported in other studies (Castilla, Pope & Jha, 2016; Klein, Desassis & Oddou‐Muratorio, 2008; Setsuko, Nagamitsu & Tomaru, 2013; Tambarussi et al., 2015), but see also Tarazi, Sebbenn, Kageyama and Vencovsky (2013). The high fertility of trees associated with greater dbh values may be related to the large size of the crowns and the high flower production level, which are favored by foraging pollinators (Setsuko et al., 2013).

### 4.7 Outcrossing and inbreeding

Our results show that the species presents a mixed mating system, with a predominance of outcrossing (>75%) and individual variation among trees (0.53–1.0). *Hymenaea stignocarpa* is monoecious and self‐compatible, and seeds can be produced through mixtures of outcrossing and selfing (Gibbs et al., 1999; Moraes & Sebbenn, 2011). Plants with a mixed mating system have evolutionary advantages over strictly selfing or outcrossing species due to the possibilities for gene recombination through both seed production mechanisms. These processes may guarantee reproduction in specific situations, such as the spatial isolation of trees (Moraes & Sebbenn, 2011). For *H. stignocarpa*, self‐fertilization occurs due to the mobility and food foraging habits of bats as they move between flowers of the same tree before flying to other trees (Collevatti, Estolano, Garcia & Hay, 2009). The outcrossing (or selfing) variations among trees can be explained by individual variations in inbreeding depression for inbred seeds produced through selfing and mating among relatives. For selfing, this variation depends on the genetic load (deleterious alleles) of the mother; for mating among related trees (*t*_r_), it depends on the mother and father and the probability that ovules and pollen gametes carrying deleterious alleles are combined in homozygosis during fertilization. Inbreeding from both selfing and mating among relatives will not produce inbreeding depression if the parents do not present deleterious alleles or if the alleles are combined in a heterozygous state. In contrast, inbreeding depression will be high for parent trees with an increased number of deleterious alleles, and many inbred seeds will not germinate (and, as such, will not be included in genetic analyses). Thus, variations in the genetic load of trees can result in individual variations in the outcrossing rate due to inbreeding depression, which may result in the mortality of some offspring.

Our estimate of group coancestry (Θ) indicates that under conditions of random mating in PA and PF, low levels of inbreeding should be expected in the descendant populations (<1%). This result is supported by the results for PF juveniles, which detected no inbreeding, although we did detect a 4.1% rate of realized mating among related individuals (*t*_r_). In contrast, the offspring of both populations presented inbreeding due to selfing and mating among relatives. Both paternity and mixed mating system (MLTR) analyses showed that mating among related trees was higher in PA than PF. The SGS in adults and the IBD pollen dispersal pattern of the populations can explain the results for mating among related trees (*t*_r_) and the differences between populations. The mean distance at which mating among related trees (*t*_r_) was detected in PA (272 m) is similar to the detected distance of SGS (250 m); however, in PF, the mean distance (1,121 m) was higher than the observed SGS distance (250 m). For PF offspring, only 31% of mating among relatives occurred within 350 m, and for PF juveniles, 75% of mating among relatives occurred within this distance. These results show that because of long‐distance pollen dispersal, related adults are separated by distances greater than the SGS distance and are able to mate.

Self‐fertilization (*s*) and mating among related trees (*t*_r_) produce inbreeding in descendant generations, which may result in inbreeding depression. Inbreeding was only detected for offspring (minimum of 0.35), suggesting selection against inbred individuals in the offspring, juvenile, and adult life stages. We also detected lower levels of selfing than mating among related trees (*t*_r_). Selfing produces a minimum of 50% inbreeding in descendants, whereas mating among related trees (*t*_r_) produces levels of inbreeding similar to those of coancestry between the parents. The mean inbreeding estimated for selfed offspring (*F*_s_) and offspring from mating among related trees (*F*_r_) confirm these expectations, with low levels of offspring originating from mating among related trees (*t*_r_). However, these *F*_r_ values were higher than the estimated mean coancestry coefficient between assigned related parents (*θ*_r_) for offspring (PA = 0.21; PF = 0.18) and juveniles (0.17). The differences can be explained by the fact that the individual levels of inbreeding for offspring and juveniles and the pairwise coancestry between parents were measured based on the genotypic correlation coefficients of identical‐by‐descent alleles (see Ackerman et al., 2017).

### 4.8 Correlated mating

The paternity correlation was high and similar between populations (*r*_p_ > 0.5), but it was variable among seed trees. This can be explained by pollen being deposited within trees, which is associated with individual variations in flowering phenology (Moraes & Sebbenn, 2011). For both populations, this resulted in a low (<3) effective number of pollen donors fertilizing seed trees (*N*_ep_), fruits (*N*_ep(w)_), and fruits within trees (*N*_ep(a)_), with a maximum of eight pollen donors. As explained above, the amount of nectar and the nectar sugar concentration are factors that affect the number of flowers a pollinator will visit. A low effective number of pollen donors have been reported in other studies on bat‐pollinated trees, ranging from 4 to 13 (Carneiro et al., 2011; Moraes & Sebbenn, 2011; Quesada, Fuchs & Lobo, 2001).

Paternity correlation was similar within (*r*_p(w)_) and among (*r*_p(a)_) fruits, which indicates the probability of finding full‐sibs within and among fruits; however, at the level of individual trees, the *r*_p(w)_ values were generally lower than the *r*_p(a)_ values. Higher *r*_p(w)_ values than *r*_p(a)_ values have been reported for other tropical tree species (Giustina et al., 2018; Manoel et al., 2015; Quesada et al., 2001; Silva et al., 2011; Tambarussi et al., 2016; Wadt et al., 2015). Thus, as we also found self‐fertilization in both populations, and families presented mixtures of self‐sibs, half‐sibs, full‐sibs, and self‐half‐sibs, with inbred selfed offspring and substantial proportions of half‐sibs and full‐sibs presenting inbreeding due to the detected mating among related individuals. Therefore, the coancestry coefficient (Θ) was higher and the effective size (*N*_e_) within families was lower than the corresponding values expected for panmictic populations (Θ = 0.125; *N*_e_ = 4).

### 4.9 Pairwise coancestry

Estimates of pairwise coancestry between parents and descendants were sometimes lower (juveniles–mother: *θ*_1_ = 0.19; juveniles–father: *θ*_2_ = 0.19; PA: offspring–father, *θ*_2_ = 0.13) and sometimes higher (offspring–mother, PA: *θ*_1_ = 0.42; PF: *θ*_1_ = 0.32; offspring–father in PF, *θ*_2_ = 0.29) than expected (0.25). These results can be explained by the fact that estimates of pairwise relatedness between parents using gene markers and probabilistic methods such as those used here (Loiselle et al., 1995) are subjected to bias, which can result in estimates that are different than expected, especially if the number of loci is lower than 20; in our case, estimates were based on six loci (see, Ackerman et al., 2017; Moraes, Gaino, Moraes, Freitas & Sebbenn, 2012).

The estimate of mean coancestry within families from mating system indices (Θ) was significantly positive correlated (*ρ* = 0.608) with the estimated mean family pairwise coancestry within families (*θ*_w_). These estimates are based on very different approaches. The mean coancestry estimated within families from mating system indices (Θ) is determined by inbreeding in the mother trees (*F*_m_), selfing (*s* = 1 − *t*_m_), and correlated mating (*r*_p_), and it can reach a minimum coancestry coefficient of 0.125 (half‐sibs) between two sibs within a family if there is an absence of inbreeding in the mother trees (*F*_m_ = 0), selfing (*s* = 1 − *t*_m_ = 0), and correlated mating (*r*_p_ = 0) and a maximum value of 1 if there is inbreeding in the mother trees (*F*_m_ = 1) with sibs originated from selfing (*s* = 1 − *t*_m_ = 1). In contrast, the estimate of pairwise coancestry between two sibs from the same family based on the Loiselle et al. (1995) method, which was derived as a correlation coefficient of coancestry, can present pairwise values ranging from −1 to 1. Obviously, the Loiselle et al. (1995) method is also affected by inbreeding in the mother trees (*F*_m_), selfing (*s* = 1 − *t*_m_), and correlated mating (*r*_p_), as these indices increase the frequency of identity‐by‐decent alleles within families. However, the Loiselle et al. (1995) method is probabilistic, and even if individuals are related, *θ*_w_ values may be different than expected and in some cases can be negatives (see Ackerman et al., 2017; Moraes et al., 2012), resulting in underestimates in the pairwise coancestry coefficient. Due to this, Moraes et al. (2012) have suggested that coancestry within families must be preferentially estimated by mating system indices, especially to determine the variance effective size within families.

### 4.10 Inbreeding depression

Our study shows strong evidence of inbreeding depression (ID) for *H. stigonocarpa*. Inbreeding was detected only in offspring, which we can attribute to selfing and mating among relatives. However, for juveniles, no selfed individuals were detected, mating among related trees (*t*_r_) was low (4.1%), and no inbreeding was detected. Again, these results suggest selection against inbred individuals between the seed and juvenile stages. In the provenance and progeny test, we observed a high rate of aborted seeds and a high mortality. Progeny from PA showed lower levels of survival (54%) than those from PF (64%), and the levels of inbreeding in PA were also higher. Survival and mean height (*H*) were greater for offspring originating from mating among unrelated individuals (*t*_u_) than those originating from selfing (*s*) and mating among relatives (*t*_r_). The ID for survival and H were higher for offspring produced from selfing (*s*) than offspring produced from mating among related trees (*t*_r_) in both populations. These results confirm the expectation that selfing results in more ID than mating among relatives due to a greater probability that identical‐by‐descent alleles are combined in a homozygous state in the selfed offspring. Nevertheless, *H* was less affected by inbreeding than survival.

### 4.11 Implications for conservation genetics

The two investigated populations are inserted within a large savannah landscape (approximately 2,523 ha) composed of a mix of pastures and sugarcane and eucalyptus plantations and interspersed with small forest fragments and isolated *H. stigonocarpa* trees in pastures. The PA population consists of an area of 109.12 ha of isolated trees in a pasture located approximately 5 km from the PF population, which is within a large forest fragment (666.7 ha). Our results show that these two populations are not reproductively and genetically isolated, as there is pollen and seed flow between both populations and immigration from outside the study area. As the isolated trees of the PA population presented higher allelic richness than the trees of the fragmented PF population, for in situ conservation, it is important to conserve both areas to maintain the genetic diversity of the neighboring populations. Our results also indicate that populations of *H. stigonocarpa* must be preserved at distances of at least 5 km to maintain genetic connectivity through gene flow, which is carried out by both pollen and seed dispersal.

For ex situ conservation, our results indicate that (a) as there are no strong genetic differences between populations, seeds can be collected from just one or both populations; (b) due to the presence of similar levels of SGS in both populations and to avoid the collection of seeds from related trees, we recommend harvesting seeds from trees at least 250 m apart; (c) as the paternity correlation among and within fruits was similar and high, indicating a low number of effective pollen donors fertilizing the trees and their fruits, seed collection should include many fruits from each tree to increase the probability of more pollen donors contributing to the seed samples. Furthermore, we suggest mixing seeds from different seed trees in equal proportions (maternal gamete control) to decrease the variation in the maternal genetic contribution of seeds used in conservation and reforestation plans; (d) to retain an effective size of 150 in progeny array samples, seed collection must include at least 83 trees from PA and 78 from PF. However, because we detected inbreeding depression for survival due to a high number of inbred seeds, we suggest collecting a greater number of seeds from each tree and selecting seedlings that show greater vigor and growth at the nursery stage for ex situ conservation, tree improvement, and environmental reforestation to maximize the survival rates. Finally, the species presents a mixed mating system combined with high levels of correlated mating, resulting in a coancestry within families (Θ) similar to that expected for full‐sibs (Θ = 0.25); thus, for tree improvement programmes, we suggest estimating the additive genetic variance ($\sigma_{A}^{2}$) as $\sigma_{A}^{2}=\sigma_{f}^{2}/2\Theta =\sigma_{f}^{3}/0.504$ ($\sigma_{f}^{2}$ is the genetic variance among families), instead of using the general formula for half‐sib families ($\sigma_{A}^{2}=\sigma_{f}^{3}/0.25$).

## CONFLICT OF INTEREST

None declared.

## AUTHOR CONTRIBUTIONS

This paper is the result of a research project supported by Conselho Nacional de Desenvolvimento Científico e Tecnológico (CNPq; Project 481039/2010‐4). This study was part of the PhD thesis of Marcela A. Moraes. Alexandre M. Sebbenn, Mario L.T. Moraes, and Miguel L.M. Freitas conceived and designed the study. Marcela A. Moraes, Thaisa Y.K. Kubota performed the field and laboratory work. Alexandre M. da Silva and Jose Cambuim performed the fieldwork and Alexandre M. da Silva did the Figure 1. Mario L.T. Moraes, Celso L. Marino, and Bruno C. Rossini contributed reagents, materials, and laboratory facilities. Alexandre M. Sebbenn and Marcela A. Moraes conducted the analyses on the data, wrote the paper, and received feedback from all co‐authors.
