# Supplementary material for: Long‐distance pollen and seed dispersal and inbreeding depression in Hymenaea stigonocarpa (Fabaceae: Caesalpinioideae) in the Brazilian savannah
Source: Ecol Evol. 2018 Jul 13;8(16):7800–16. doi: 10.1002/ece3.4253 (PMC6144967; doi:10.1002/ece3.4253)
Supplement: Supplementary file 1 [file ECE3-8-7800-s001.doc]

**Supplementary material: Table S1** Mean, minimum and maximum pairwise coancestry between offspring within families ($\theta_{w}$) of PA and PF populations

| Tree | $n_{p}$ | $\theta_{w}$ (SD) | Min/Max | Tree | $n_{p}$ | $\theta_{w}$ (SD) | Min/Max |
| --- | --- | --- | --- | --- | --- | --- | --- |
| PA11 | 351 | 0.304 (0.129) | -0.036/0.700 | PF610 | 153 | 0.489 (0.165) | 0.123/0.861 |
| PA15 | 435 | 0.454 (0.122) | 0.148/0.850 | PF700 | 435 | 0.202 (0.113) | -0.060/0.562 |
| PA24 | 171 | 0.369 (0.115) | 0.073/0.656 | PF702 | 406 | 0.309 (0.149) | -0.046/0.771 |
| PA34 | 153 | 0.420 (0.146) | 0.093/0.757 | PF703 | 406 | 0.139 (0.164) | -0.234/0.645 |
| PA37 | 435 | 0.428 (0.149) | 0.071/0.901 | PF704 | 435 | 0.481 (0.135) | 0.132/0.890 |
| PA237 | 120 | 0.276 (0.128) | 0.043/0.630 | PF707 | 435 | 0.273 (0.153) | -0.094/0.727 |
| PA240 | 276 | 0.321 (0.136) | 0.031/0.778 | PF710 | 406 | 0.335 (0.182) | -0.023/0.927 |
| PA249 | 406 | 0.493 (0.131) | 0.211/0.831 | PF712 | 435 | 0.214 (0.124) | -0.097/0.710 |
| PA256 | 276 | 0.370 (0.119) | 0.123/0.728 | PF714 | 300 | 0.474 (0.145) | 0.161/0.882 |
| PA262 | 91 | 0.355 (0.125) | 0.041/0.630 | PF715 | 435 | 0.339 (0.143) | -0.150/0.810 |
| PA272 | 253 | 0.276 (0.110) | -0.004/0.562 | PF716 | 435 | 0.159 (0.152) | -0.245/0.611 |
| PA292 | 190 | 0.394 (0.138) | 0.078/0.806 | PF721 | 435 | 0.170 (0.147) | -0.190/0.697 |
| PA299 | 120 | 0.630 (0.139) | 0.335/1.126 | PF722 | 435 | 0.350 (0.116) | 0.045/0.779 |
| PA313 | 378 | 0.493 (0.140) | 0.173/0.864 | PF723 | 300 | 0.400 (0.128) | -0.013/0.713 |
| PA321 | 171 | 0.358 (0.133) | 0.037/0.780 | PF724 | 276 | 0.529 (0.163) | 0.125/0.989 |
| PA334 | 210 | 0.312 (0.145) | -0.089/0.670 | Total | 5727 | 0.306 (0.188) | -0.245/0.989 |
| PA339 | 171 | 0.411 (0.131) | 0.094/0.753 |  |  |  |  |
| PA342 | 378 | 0.295 (0.141) | -0.068/0.706 |  |  |  |  |
| PA358 | 435 | 0.509 (0.169) | 0.045/0.934 |  |  |  |  |
| PA368 | 231 | 0.349 (0.134) | -0.073/0.689 |  |  |  |  |
| Total | 5251 | 0.398 (0.161) | -0.089/1.126 |  |  |  |  |

$n_{p}$ is the number of pairwise individuals; SD is the standard deviation, Min/Max is the minimum and maximum (Min/Max) pairwise coancestry

**Supplementary material: Table S2** Estimates of genetic diversity and mating system indices for each family in PA and PF populations

| Tree | $F_{m}$ |  | $R$ | $H_{o}$ | $F_{o}$ | $t_{m}$(SD) | $t_{m}-t_{s}$(SD) | $r_{p}$(SD) | $r_{p(w)}$(SD) | $r_{p(a)}$(SD) | $N_{ep}$ | $N_{ep(w)}$ | $N_{ep(a)}$ | $\Theta$ | $N_{e}$ |
| --- | --- | --- | --- | --- | --- | --- | --- | --- | --- | --- | --- | --- | --- | --- | --- |
| PA11 | 0.15 | 27 | 5.2 | 0.44 | 0.22 | 0.96 (0.00) | 0.18 (0.03) | 0.12 (0.03) | 0.12 (0.07) | 0.11 (0.02) | 8.5 | 8.1 | 8.9 | 0.171 | 2.67 |
| PA15 | 0.23 | 30 | 2.8 | 0.24 | 0.39 | 0.58 (0.01) | 0.21 (0.06) | 0.49 (0.13) | 1.00 (0.47) | 0.31 (0.11) | 2.0 | 1.0 | 3.3 | 0.335 | 1.44 |
| PA24 | 0.04 | 19 | 4.8 | 0.35 | 0.28 | 0.90 (0.00) | 0.20 (0.07) | 0.25 (0.06) | 0.44 (0.21) | 0.16 (0.04) | 4.1 | 2.3 | 6.2 | 0.184 | 2.41 |
| PA34 | 0.44 | 18 | 4.9 | 0.37 | 0.22 | 0.94 (0.00) | 0.25 (0.06) | 0.21 (0.04) | 0.37 (0.18) | 0.12 (0.01) | 4.9 | 2.7 | 8.6 | 0.235 | 1.95 |
| PA37 | -0.10 | 30 | 4.4 | 0.38 | 0.25 | 0.68 (0.00) | 0.23 (0.05) | 0.26 (0.06) | 0.50 (0.24) | 0.18 (0.04) | 3.8 | 2.0 | 5.6 | 0.234 | 2.02 |
| PA237 | 0.15 | 16 | 4.5 | 0.49 | 0.10 | 0.88 (0.00) | 0.14 (0.08) | 0.13 (0.02) | 0.25 (0.13) | 0.10 (0.00) | 7.6 | 4.0 | 10.0 | 0.195 | 2.31 |
| PA240 | -0.10 | 24 | 4.6 | 0.38 | 0.31 | 0.88 (0.00) | 0.31 (0.06) | 0.36 (0.07) | 0.50 (0.23) | 0.27 (0.06) | 2.8 | 2.0 | 3.7 | 0.192 | 2.37 |
| PA249 | 0.25 | 29 | 3.8 | 0.42 | 0.09 | 0.80 (0.00) | 0.36 (0.04) | 0.49 (0.08) | 0.70 (0.33) | 0.34 (0.09) | 2.0 | 1.4 | 2.9 | 0.275 | 1.76 |
| PA256 | 0.20 | 24 | 5.1 | 0.52 | 0.05 | 0.99 (0.01) | 0.35 (0.05) | 0.37 (0.07) | 0.48 (0.21) | 0.27 (0.06) | 2.7 | 2.1 | 3.6 | 0.207 | 2.27 |
| PA262 | 0.16 | 14 | 3.8 | 0.41 | 0.14 | 0.87 (0.00) | 0.15 (0.10) | 0.23 (0.08) | 0.73 (0.35) | 0.10 (0.00) | 4.4 | 1.4 | 10.0 | 0.211 | 2.11 |
| PA272 | 0.09 | 23 | 4.2 | 0.38 | 0.18 | 0.92 (0.00) | 0.18 (0.06) | 0.27 (0.06) | 0.38 (0.18) | 0.21 (0.05) | 3.7 | 2.6 | 4.9 | 0.192 | 2.39 |
| PA292 | 0.56 | 20 | 4.3 | 0.37 | 0.14 | 0.86 (0.00) | 0.23 (0.06) | 0.22 (0.05) | 0.46 (0.22) | 0.14 (0.03) | 4.6 | 2.2 | 7.1 | 0.287 | 1.66 |
| PA299 | 0.44 | 16 | 4.1 | 0.35 | 0.17 | 0.72 (0.02) | 0.22 (0.06) | 0.23 (0.08) | 0.93 (0.43) | 0.13 (0.03) | 4.3 | 1.1 | 7.6 | 0.316 | 1.50 |
| PA313 | 0.39 | 28 | 3.6 | 0.32 | 0.25 | 0.69 (0.00) | 0.31 (0.04) | 0.47 (0.08) | 0.71 (0.33) | 0.33 (0.09) | 2.1 | 1.4 | 3.1 | 0.338 | 1.44 |
| PA321 | 0.40 | 19 | 3.3 | 0.32 | 0.23 | 0.76 (0.01) | 0.16 (0.08) | 0.22 (0.07) | 0.53 (0.25) | 0.16 (0.05) | 4.5 | 1.9 | 6.3 | 0.294 | 1.61 |
| PA334 | 0.04 | 21 | 3.8 | 0.43 | 0.18 | 0.73 (0.01) | 0.14 (0.08) | 0.25 (0.07) | 0.54 (0.26) | 0.17 (0.04) | 4.1 | 1.8 | 6.0 | 0.226 | 2.05 |
| PA339 | 0.10 | 19 | 3.2 | 0.40 | 0.18 | 0.53 (0.02) | 0.05 (0.09) | 0.22 (0.10) | 1.00 (0.48) | 0.17 (0.06) | 4.5 | 1.0 | 6.0 | 0.306 | 1.56 |
| PA342 | 0.05 | 28 | 4.2 | 0.50 | 0.06 | 0.62 (0.01) | 0.09 (0.06) | 0.12 (0.02) | 0.13 (0.08) | 0.11 (0.01) | 8.7 | 7.9 | 9.0 | 0.257 | 1.88 |
| PA358 | 0.21 | 30 | 3.4 | 0.43 | 0.13 | 0.61 (0.01) | 0.25 (0.03) | 0.36 (0.08) | 0.54`(0.26) | 0.28 (0.09) | 2.8 | 1.9 | 3.5 | 0.312 | 1.56 |
| PA368 | -0.10 | 22 | 4.7 | 0.53 | 0.03 | 0.83 (0.00) | 0.23 (0.07) | 0.27 (0.07) | 0.74 (0.34) | 0.12`(0.02) | 3.7 | 1.4 | 8.1 | 0.196 | 2.38 |
| PF610 | -0.10 | 18 | 4.6 | 0.41 | 0.23 | 0.89 (0.00) | 0.31 (0.07) | 0.36 (0.09) | 0.80 (0.36) | 0.24 (0.07) | 2.8 | 1.3 | 4.2 | 0.189 | 2.35 |
| PF700 | -0.10 | 30 | 4.5 | 0.42 | 0.20 | 0.85 (0.01) | 0.00 (0.03) | 0.12 (0.02) | 0.14 (0.07) | 0.11 (0.01) | 8.4 | 6.9 | 9.4 | 0.175 | 2.64 |
| PF702 | -0.10 | 29 | 3.6 | 0.35 | 0.28 | 0.67 (0.00) | 0.09 (0.08) | 0.27 (0.06) | 0.44 (0.21) | 0.20 (0.05) | 3.7 | 2.3 | 5.0 | 0.237 | 1.99 |
| PF703 | -0.10 | 29 | 3.9 | 0.44 | 0.24 | 1.00 (0.00) | 0.17 (0.04) | 0.21 (0.04) | 0.18 (0.09) | 0.20 (0.04) | 4.8 | 5.6 | 4.9 | 0.151 | 2.98 |
| PF704 | 0.13 | 30 | 2.9 | 0.37 | 0.04 | 0.58 (0.00) | 0.16 (0.06) | 0.38 (0.08) | 0.69 (0.32) | 0.27 (0.08) | 2.6 | 1.4 | 3.7 | 0.269 | 1.80 |
| PF707 | 0.00 | 30 | 3.8 | 0.40 | 0.24 | 0.61 (0.00) | 0.04 (0.08) | 0.13 (0.02) | 0.20 (0.10) | 0.11 (0.01) | 7.6 | 5.0 | 9.2 | 0.247 | 1.93 |
| PF710 | 0.40 | 29 | 3.2 | 0.35 | 0.16 | 0.63 (0.00) | 0.11 (0.05) | 0.21 (0.05) | 0.38 (0.18) | 0.16 (0.04) | 4.7 | 2.6 | 6.1 | 0.345 | 1.42 |
| PF712 | 0.04 | 30 | 4.2 | 0.55 | 0.05 | 0.93 (0.00) | 0.16 (0.04) | 0.16 (0.04) | 0.16 (0.09) | 0.16 (0.03) | 6.1 | 6.4 | 6.4 | 0.167 | 2.79 |
| PF714 | 0.01 | 25 | 3.4 | 0.47 | 0.10 | 0.96 (0.00) | 0.29 (0.04) | 0.66 (0.07) | 0.87 (0.39) | 0.49 (0.10) | 1.5 | 1.2 | 2.0 | 0.215 | 2.19 |
| PF715 | -0.20 | 30 | 3.6 | 0.50 | 0.05 | 0.71 (0.00) | 0.17 (0.07) | 0.38 (0.10) | 0.77 (0.36) | 0.26 (0.08) | 2.7 | 1.3 | 3.9 | 0.233 | 2.06 |
| PF716 | 0.20 | 30 | 3.6 | 0.44 | 0.19 | 0.95 (0.01) | 0.09 (0.04) | 0.30 (0.05) | 0.39 (0.19) | 0.23 (0.05) | 3.3 | 2.5 | 4.3 | 0.173 | 2.68 |
| PF721 | 0.10 | 30 | 3.4 | 0.50 | 0.03 | 0.82 (0.01) | 0.04 (0.06) | 0.24 (0.06) | 0.29 (0.15) | 0.20 (0.05) | 4.3 | 3.4 | 5.1 | 0.195 | 2.43 |
| PF722 | 0.00 | 30 | 3.2 | 0.45 | 0.02 | 0.90 (0.00) | 0.26 (0.05) | 0.60 (0.08) | 0.82 (0.38) | 0.42 (0.09) | 1.7 | 1.2 | 2.4 | 0.211 | 2.26 |
| PF723 | 0.00 | 25 | 4.2 | 0.52 | 0.01 | 0.92 (0.00) | 0.32 (0.05) | 0.43 (0.08) | 0.57 (0.26) | 0.32 (0.08) | 2.3 | 1.8 | 3.1 | 0.192 | 2.44 |
| PF724 | 0.24 | 24 | 3.5 | 0.43 | 0.16 | 0.80 (0.00) | 0.23 (0.04) | 0.37 (0.07) | 0.59 (0.28) | 0.26 (0.07) | 2.7 | 1.7 | 3.8 | 0.260 | 1.83 |

$F_{m}$ and $F_{o}$ are the fixation index for seed trees and within families, respectively; is sample size; $R$ is the allelic richness for 14 individuals genotyped for all the used six loci; $H_{o}$ is the observed heterozygosity; $t_{m}$ is the multilocus outcrossing rate; $t_{m}-t_{s}$ is the rate of mating among related individuals; $r_{p}$, $r_{p(w)}$, and $r_{p(a)}$ are the paternity correlations within and among, within, and among fruits, respectively; $N_{ep}$, $N_{ep(w)}$, and $N_{ep(a)}$ are the effective numbers of pollen donors within and among, within, and among fruits, respectively; $\Theta$and $N_{e}$ are the coancestry and effective size within families, respectively; SD is the standard error

52

**Supplementary material: Table S3** Spearman rank correlation ($\rho$) between the sample size ($n$), allelic richness ($R$), observed heterozygosity ($H_{o}$), fixation index ($F_{o}$), outcrossing rate ($t_{m}$), rate of selfing and mating among relatives [$s+(t_{m}-t_{s})$], effective number of pollen donors ($N_{ep}$), and variance effective size within families ($N_{e}$); * *P*< 0.05; ** *P*< 0.01

|  | $H_{o}$ | $F$ | $s+(t_{m}-t_{s})$ | $N_{ep}$ | $N_{e}$ |
| --- | --- | --- | --- | --- | --- |
| $n$ | 0.183 | -0.107 | 0.128 | -0.192 | 0.082 |
| $R$ | 0.228 | 0.123 | -0.410* | 0.373* | 0.515** |
| $H_{o}$ |  | -0.698** | -0.411* | 0.016 | 0.601** |
| $F$ |  |  | 0.092 | 0.169 | -0.123 |
| $s+(t_{m}-t_{s})$ |  |  |  | -0.399* | -0.819** |
| $N_{ep}$ |  |  |  |  | 0.172 |
